# A Retrospective Analysis of Culture-Confirmed Enterococci Bloodstream Infections in South Africa, 2016–2020: A Cross-Sectional Study

**DOI:** 10.3390/tropicalmed8010019

**Published:** 2022-12-27

**Authors:** Ruth Mogokotleng, Husna Ismail, Olga Perovic, Sabelle Jallow

**Affiliations:** 1 Centre for Healthcare-Associated Infections, Antimicrobial Resistance and Mycoses, National Institute for Communicable Diseases of National Health Laboratory Service, Johannesburg 2192, South Africa; 2 Department of Clinical Microbiology and Infectious Diseases, School of Pathology, Faculty of Health Sciences, University of Witwatersrand, Johannesburg 2000, South Africa

**Keywords:** enterococci, ESKAPE, antimicrobial resistance (AMR), bloodstream infections (BSI), South Africa

## Abstract

(1) **Background**: The emergence of multidrug resistance enterococci is a major public health concern. This study aimed to determine the prevalence and antimicrobial resistance of enterococci isolated from blood cultures over a five-year period (2016–2020) at public hospitals in South Africa. (2): **Methods**: A retrospective analysis of clinical enterococci isolated from bloodstream infection samples at the South African public hospitals was conducted. The ESKAPE dataset from January 2016 to December 2020 was obtained from the central data warehouse (CDW) at the National Health Laboratory Service (NHLS). (3): **Results**: Following de-duplication, a total of 130,352/306,592 organisms isolated from blood cultures were identified as ESKAPE pathogens. In this study, *K. pneumoniae* (25%; 33,082/130,352), was the most frequently isolated pathogen from blood cultures, followed by *S. aureus* (23%; 29,922/130,352) and enterococci (16%; 21,339/130,352). Of the enterococci cases, about 43% (9132/21,339) of cases were from the infants aged (<1-year old) and 32% (6745/21,339) from the adult patients. No changes observed in vancomycin, teicoplanin, and linezolid susceptibility; however, *E. faecium* and *E. faecalis* blood culture isolates remained highly susceptible (>97%) to these antibiotics. (4): **Conclusions**: The current study revealed a significant increase of *E. faecalis* and *E. faecium* blood culture isolates as compared to the previous national ESKAPE data. Low vancomycin resistance was observed. Continuous monitoring of antimicrobial resistant *Enterococcus* species is warranted in South Africa.

## 1. Introduction

The emergence and spread of antimicrobial resistance (AMR) among bacteria cause significant morbidity, mortality, and economic burden [1,2]. Healthcare-associated pathogens (HAPs) are an ongoing medical issue, having the biggest impact on healthcare system due to their multidrug resistance against important and critical antimicrobial classes such as β-lactams, aminoglycosides, glycopeptides, fluoroquinolones, polymyxins, and penicillins [3,4]. The ESKAPE pathogens (i.e., *Enterococcus faecium*, *Staphylococcus aureus*, *Klebsiella pneumoniae*, *Acinetobacter baumannii*, *Pseudomonas aeruginosa*, and *Escherichia coli* are important HAPs increasingly associated with healthcare-associated infections (HAIs) and are life-threatening, particularly among the critically ill and immunocompromised patients [1,5,6,7,8,9].

*Enterococcus* species are important opportunistic organisms capable of causing different clinical manifestations including bloodstream infections (BSIs), endocarditis, neonatal sepsis, urinary tract, wound, and intra-abdominal infections [3,10]. These organisms usually exhibit resistance against important antimicrobial classes of antibiotics through intrinsic and acquired mechanisms [6,7,8].

Multidrug-resistant enterococci are critical health issues of which vancomycin-resistant enterococci (VRE) have emerged as the highly priority pathogen [11,12,13,14]. In South Africa, VREs are regarded as a category 4 notifiable medical condition, associated with nosocomial outbreaks and high mortality rates [15]. Infections caused by VREs are difficult to manage due to their persistence against common and critical therapeutic antimicrobial agents including ampicillin and glycopeptides (e.g., vancomycin) [6]. Moreover, enterococcal BSIs carry significant morbidity in both hospitalized and community-dwelling patients, with an attributable mortality rate of 26%, reaching 37% in the event of infections caused by vancomycin-resistant enterococci (VRE) [16,17].

Ampicillin remains the first-line drug of choice for *E. faecalis*, but not for *E. faecium*, which is intrinsically resistant to ampicillin [18]. Vancomycin and teicoplanin are glycopeptides that exhibit activity against both *E. faecium* and *E. faecalis* [19]. Linezolid is a watch and reserve drug currently approved by the Food and Drug Administration (FDA) to treat serious infections caused by VRE [20]. Several published studies have demonstrated a rapid increase in the burden of enterococcal BSIs and a shift towards more resistant strains [21,22]. Monitoring the prevalence and antimicrobial resistance trends of enterococci causing BSIs provides critically important information for clinicians as well as informing treatment strategies, infection control and prevention (IPC) programs, policy formulation, and emerging resistance threats. In this study, a retrospective analysis was conducted to determine the prevalence and described the epidemiology of enterococci isolated from blood cultures at the South African public hospitals over a five-year period, from 1 January 2016 to 31 December 2020.

## 2. Materials and Methods

### 2.1. Study Design, Population and Setting

We conducted a cross-sectional analysis of culture-confirmed enterococci BSI data obtained from the central data warehouse (CDW) at the National Health Laboratory Service from 1 January 2016 to 31 December 2020. The National Health Laboratory Service (NHLS) comprises a network of pathology laboratories that process clinical specimens from public hospitals across South Africa, serving an estimated 80% of the country’s population. The extracted dataset included records from 226 pathology laboratories across all nine provinces.

The study population included all patients who had a blood culture submitted to the National Health Laboratory Service in South Africa. Positive blood cultures with an ESKAPE pathogen were included in this study.

### 2.2. Inclusion Criteria

Any case of bacterial BSI, was defined as a person, who had a blood culture from which any of the ESKAPE pathogens was isolated at a NHLS microbiology laboratory.

### 2.3. Exclusion Criteria

Positive blood cultures with the same organism, which had been collected within 21 days of a first positive blood culture, were regarded as duplicates and excluded from this analysis.

### 2.4. Laboratory Procedures

All blood culture samples isolated from patients with BSI were collected aseptically from the public hospitals and sent to the inhouse NHLS diagnostic microbiology laboratory for processing. Identification of pathogens was performed on automated systems (i.e., Vitek-2 (bioMérieux, Marcy-l’Étoile, France), Microscan Walkaway (Beckman Coulter, Brea, CA, USA), or mass spectrometry instruments such as Vitek MS (bioMérieux)) [23]. Antimicrobial susceptibility testing was performed using Vitek-2 or MicroScan walkaway instrument, and results were interpreted according to the Clinical and Laboratory Standards Institute recommendations [23].

### 2.5. Data Source

Blood culture (culture-positive and culture-negative blood culture) data from 1 January 2016 to 31 December 2020 were obtained from the central data warehouse (CDW) at the NHLS. Data extracted included patient identifying information, demographics, facility name, province in which the facility is located, specimen type, date of specimen collection, tests requested, culture results (organism identification for positive cultures), and antimicrobial susceptibility results.

### 2.6. Data Analysis

Blood culture data were cleaned and analyzed using Stata version 15.1. Categorical variables were presented as tables, and the chi-squared test was used to compare differences between variables. Continuous variables were summarized using median and either range or interquartile range (IQR). A *p*-value of <0.05 was considered as statistically significant.

## 3. Results

### 3.1. Patient Demographic and Clinical Characteristics

The majority of patients were from Gauteng province with 44% (9415/21,339) followed by KwaZulu-Natal 21% (4576/21,339) (Table 1); and females accounted for 48% (10,319/21,339) of cases. The median age of patients was 2 years old (range 0–115 years); about 43% (9132/21,339) of cases were infants aged <1-year old, and 32% (6745/21,339) were adult patients aged 18 to 64 years old (Table 1).

### 3.2. Cases of ESKAPE Bloodstream Infections in South Africa

**Figure 1:**
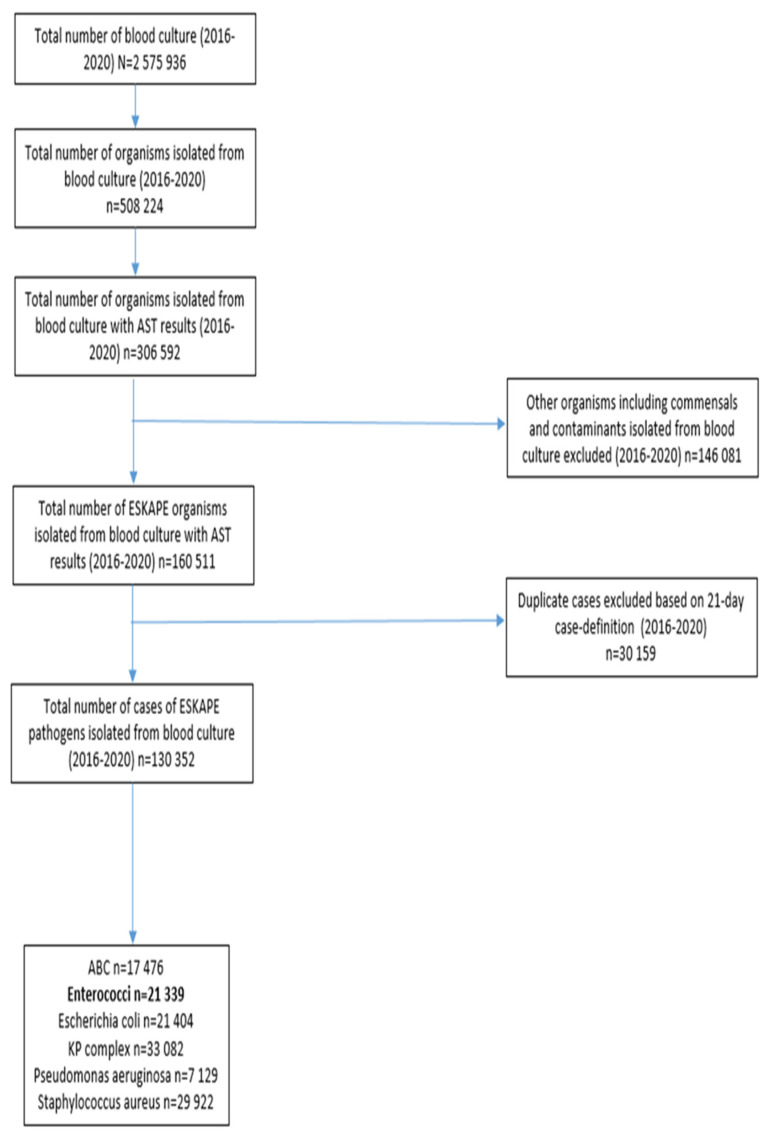
Breakdown of blood cultures submitted to the National Health Laboratory Service microbiology laboratories in South Africa, 2016–2020 (N = 2,575,936). ESKAPE: *Acinetobacter baumannii* complex (ABC): *A. baumannii*, *A. baumannii* complex; *Enterococci*: *E. avium*, *E. casselifalvus*, *E. cecorum*, *E. columbae*, *E. durans*, *E. faecalis*, *E. faecium*, *E. gallinarum*, *E. hirae*, *E. raffinosus*, other *E. species*; *Escherichia coli*; *Klebsiella pneumonia* (KP) complex: *K. pneumoniae*, *K. pneumoniae subsp ozaenae*, *K. pneumoniae subsp pneumoniae*, *K. pneumoniae subsp rhinoscleromatis*; *Pseudomonas aeruginosa*; *Staphylococcus aureus*.

**Figure 2:**
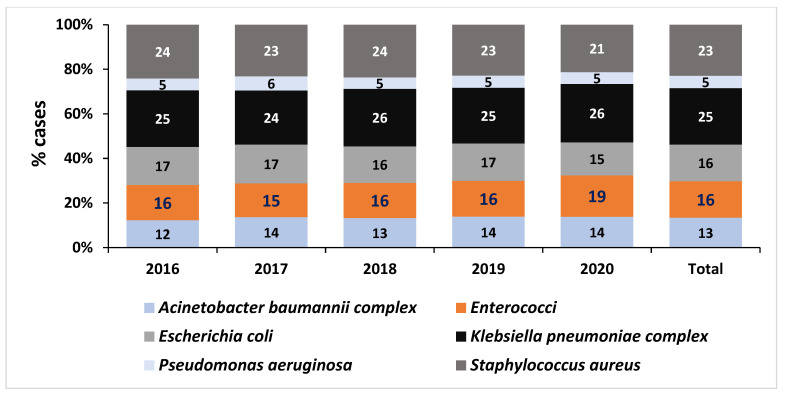
Distribution of cases with ESKAPE pathogens isolated from blood cultures in South Africa from 2016 to 2020 (*n* = 130,352). Enterococci identified included: *E. avium*, *E. casselifalvus*, *E. cecorum*, *E. columbae*, *E. durans*, *E. faecalis*, *E. faecium*, *E. gallinarum*, *E. hirae*, *E. raffinosus*, and other *E. species*. Total = represents all ESKAPE pathogens from 2016–2020.

During the 5-year period, 2,575,936 records of blood culture specimens (culture-positive and culture-negative) were extracted. Among these samples, 20% (508,224/2,575,936) had bacterial organisms present, and 60% (306,592/508,224) of these bacteria had antimicrobial susceptibility testing (AST) results. 52% (160,511/306,592) of isolated bacteria with AST results were ESKAPE pathogens. Following de-duplication and exclusion of contaminants, 130,352 cases with an ESKAPE pathogen were identified and included in the analysis. Sixteen percent (21,339/130,352) of cases reported having an enterococci isolated (Figure 1). Overall, *K. pneumoniae* (25%; 33,082/130,352), was the most frequently isolated pathogen from blood cultures, followed by *S. aureus* (23%; 29,922/130,352), *Enterococcus* species (16%; 21,339/130,352), *E. coli* (16%; 21,404/130,352), *A. baumannii* (13%; 17,476/130,352) and *P. aeruginosa* (5%; 729/130,352) (Figure 2).

### 3.3. Cases of Enterococci Bloodstream Infections in South Africa

**Figure 3:**
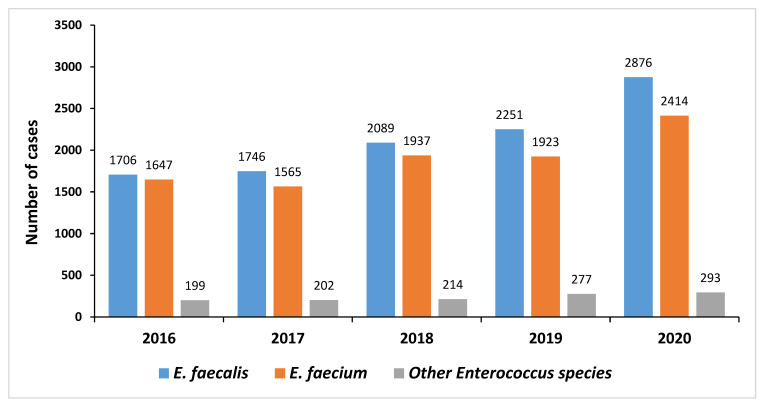
Distribution of cases with *Enterococcus* species isolated from blood cultures in South Africa from 2016 to 2020 (*n* = 21,339). Other enterococci included: *E. avium*, *E. casselifalvus*, *E. cecorum*, *E. columbae*, *E. durans*, *E. gallinarum*, *E. hirae*, *E. raffinosus*, and *E. species*.

An increase was observed in the percentage of enterococci cases in South Africa from 16% (3552/22,560) in 2016 to 19% (5583/30,044) in 2020 (*p* < 0.01) (Figure 2 and Figure 3). The increase in both *E. faecalis* (*p* = 0.016) and *E. faecium* (*p* = 0.007) cases over the five-year study period was significant (Figure 3). Of the 21,339 enterococci cases, *E. faecalis* accounted for 50% (10,668/21,339), *E. faecium* accounted for 45% (9486/21,339), while 5% (1185/21,339) were identified as other enterococci (*E. avium*, *E. casselifalvus*, *E. cecorum*, *E. columbae*, *E. durans*, *E. faecalis*, *E. faecium*, *E. gallinarum*, *E. hirae*, *E. raffinosus*).

### 3.4. Antimicrobial Susceptibility Profiles of Enterococci Bloodstream Infection Isolates in South Africa

**Figure 4:**
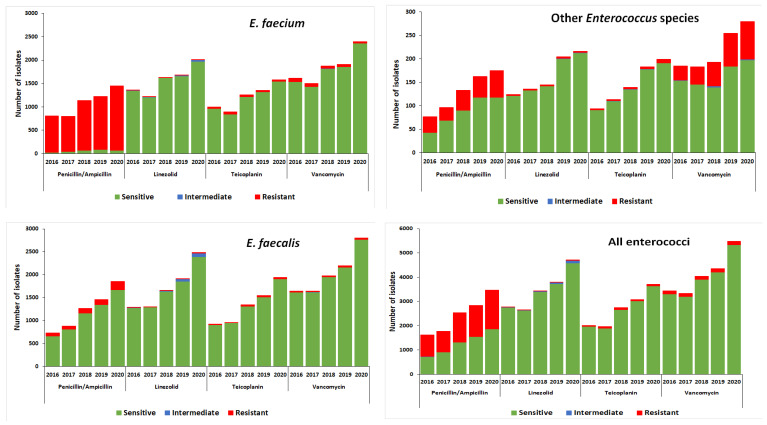
Antimicrobial susceptibility profiles for all *Enterococcus* species bloodstream infection isolates in South Africa, from 2016 to 2020 (*n* = 21,339). Other enterococci included: *E. avium*, *E. casselifalvus*, *E. cecorum*, *E. columbae*, *E. durans*, *E. gallinarum*, *E. hirae*, *E. raffinosus*, other *E. species* (*n* = 1185).

Antimicrobial susceptibility to ampicillin was similar for *E. faecium* over the five-year study period; about 95% of isolates were resistant to ampicillin (Figure 4). *E. faecium* isolates showed an increase in teicoplanin susceptibility from 97% in 2016 to 98% in 2020 (*p* < 0.01), an increase in vancomycin susceptibility from 96% in 2016 to 99% in 2020 (*p* < 0.01), and a decrease in linezolid susceptibility from 99% in 2016 to 98% in 2020 (*p* < 0.01) (Figure 4).

For *E. faecalis*, 10% of isolates were resistant to ampicillin. There were no changes to ampicillin (*p* = 0.117), vancomycin (*p* = 0.86) and teicoplanin (*p* = 0.12) susceptibility through the study period. However, susceptibility to linezolid decreased from 99% in 2016 to 96% in 2020 (*p* < 0.01).

For other enterococci, 32% of isolates were resistant to ampicillin. No changes were observed for linezolid and teicoplanin susceptibility through the study period. However, susceptibility to vancomycin decreased from 83% in 2016 to 75% in 2020 (*p* < 0.01) (Figure 4).

## 4. Discussion

Our study was conducted to determine the prevalence and AMR patterns of enterococci isolated from blood cultures at the South African public hospitals from January 2016 to December 2020. Gauteng and KwaZulu-Natal provinces accounted for the majority of isolates, probably because they are the most populous provinces with the largest hospitals. We observed a high proportion of enterococci among infants less than one year old. Other studies reported the same findings and also showed a significant association between pediatrics enterococcal infection and having a history of invasive treatment procedure/chronic illness, admission, or hospitalization [24]. However, our study did not explore such an association due to the absence of patient clinical data.

Among the ESKAPE pathogens studied, *K. pneumoniae* was the most predominantly isolated from blood cultures, followed by *S. aureus* and enterococci. Enterococci, along with *E. coli*, were the third most common pathogens isolated from blood cultures. We observed an increase in the percentage of cases of enterococci in South Africa, from 16% to 19%, when compared to other ESKAPE pathogens. The breakdown of IPC measures and infrastructure human resources within our healthcare settings during the first wave of the COVID-19 pandemic could have resulted in the increase number of enterococci cases in 2020. Among the enterococci, a higher proportion of *E. faecalis* (50%) than *E. faecium* (45%) isolated from blood culture samples was observed. A similar species distribution was reported in India (58% of *E. faecalis* and 42% of *E. faecium*). In contrary, a study in Saudi Arabia identified 72.7% *E. faecalis* and 22.8% *E. faecium* [25].

Bacterial resistance for the commonly used antibiotics is spreading globally. In our study, *E. faecium* blood culture isolates were highly resistant to ampicillin (>95%), which is in-keeping with global distribution [18]. This is due to the intrinsic ampicillin resistance in *E. faecium* [26]. Unlike *E. faecalis*, most *E. faecium* express high-levels of resistance to ampicillin as a result of expression of low-affinity penicillin-binding proteins and/or by polymorphisms in the beta subunit of this protein [27]. *E. faecalis* remains susceptible to ampicillin, which remains the drug of choice for its treatment. Antimicrobial susceptibility to ampicillin in both *E. faecalis and E. faecium* did not differ from data described by the 2017 European Centre for Disease Prevention and Control (ECDC) [28]. Our finding is in line with the study conducted in Nigeria, which showed 100% ampicillin resistance [29]. In contrast, studies in Ethiopia (Addis Ababa and Gondar) reported ampicillin resistance in enterococci with 6.7% and 5.5%, respectively [30,31].

Vancomycin, teicoplanin, and linezolid are listed as watch and reserves drugs for the treatment of enterococcal infections. Vancomycin resistance, in our study, remains uncommon in *E. faecalis* (~1% in 2016) with no change in 2020, but seemed an emerging problem in *E. faecium*, although resistance has dropped nationally over the past 5 years, from 4.5% to 1.3% (2016 to 2020). More than 97% of our *E. faecium* and *E. faecalis* isolates remained susceptible to vancomycin teicoplanin and linezolid, and this is in accordance with data reported in Ethiopian studies (5.5% in Gondar [30], 7.5% in southern Ethiopia [32], 6.7% [29] and 6.3% [33] in Addis Ababa, and 4% in Canada [34]). The low prevalence of vancomycin in the South African public hospitals could possibly be due to the fact that the usage of vancomycin in the public sector remained relatively low and stable between 2018 to 2020 [35]. Other countries such as Spain and India reported high prevalence of vancomycin resistance (80% and 82%, respectively) [36,37]. VRE incidence rates of 75%, 23.7%, and 15.8% were reported in Egypt [32], Iran [38], and Brazil [39], respectively. The higher incidence of VRE in these countries could be due to higher usage of vancomycin.

Other enterococci, *E. avium*, *E. casselifalvus*, *E. cecorum*, *E. columbae*, *E. durans*, *E. gallinarum*, *E. hirae*, *E. raffinosus*, *E. species*), although in low numbers (Table 1), showed relatively higher resistance to vancomycin of 25% compared to *E. faecium* (3%) and *E facaelis* (1.4%). Some of these species have been reported to be intrinsically resistant to vancomycin due to the presence of the *van*C gene that produces low-level resistance to vancomycin due to inner cell-wall penicillin-binding proteins [14]. These non-faecalis and non-faecium *Enterococcus* species showed higher resistance to ampicillin (32%) and vancomycin (25%) during our study period, however, they remained highly susceptible to linezolid (98%) and teicoplanin (97%).

This report provides a comprehensive overview of the burden of enterococci at a national level in South Africa over a five-year period. In addition, our study provided insight into basic patient demographics.

Our study had several limitations. We did not have data on the molecular characteristics of the isolates, to assess if the increase in the incidence in the VRE blood cultures is related to a specific clone. We were unable to explore daptomycin susceptibility in this study, which is regarded as one of the watch and reserve antibiotics due to the antibiotic not being tested by the laboratories [40]. We did not collect clinical features or antibiotic consumption data because it was passive surveillance, and therefore no potential correlation with antibiotic resistance could be evaluated. Although this data reflects isolates from all nine provinces, data from the private health sector was not included, and the results reflect only the public sectors hospitals in South Africa.

## 5. Conclusions

Our study provides important insights into the frequency of enterococci causing bloodstream infections and changes in their antimicrobial resistance over five-year period in South Africa. The current study revealed a significant increase of *E. faecalis* and *E. faecium* blood culture isolates as compared to the previous national ESKAPE data; however, >97% of enterococci isolates remained susceptible to vancomycin, teicoplanin, and linezolid. Continuous monitoring of antimicrobial resistant *Enterococcus* species is warranted in South Africa.

## Figures and Tables

**Table 1:** Demographic characteristics of cases with enterococci isolated from blood cultures in South Africa from 2016 to 2020 (*n* = 21,339).

Characteristic	Number of Cases (*N* = 21,339)	
	*n*	%
**Year**		
2016	3552	16.7
2017	3513	16.5
2018	4240	19.9
2019	4451	20.9
2020	5583	26.2
**Sex**		
Female	10,319	48.4
Male	10,073	47.2
Unknown	947	4.4
**Age categories (in years)**		
**Median age (range)**	2 (0–115)	
<1	9132	42.8
1–5	1266	5.9
6–17	582	2.7
18–64	6745	31.6
>65	1773	8.3
Unknown	1841	8.6
**Province**		
Eastern Cape	1953	9.2
Free State	1513	7.1
Gauteng	9415	44.1
KwaZulu-Natal	4576	21.4
Limpopo	829	3.9
Mpumalanga	666	3.1
North West	535	2.5
Northern Cape	200	0.9
Western Cape	1600	7.5
Unknown	52	0.2
**Enterococci distribution**		
*E. avium*	71	0.3
*E. casselifalvus*	143	0.7
*E. cecorum*	7	0.0
*E. columbae*	5	0.0
*E. durans*	105	0.5
*E. durans/hirae*	2	0.0
*E. faecalis*	10,668	50.0
*E. faecium*	9486	44.5
*E. gallinarum*	357	1.7
*E. hirae*	67	0.3
*E. raffinosus*	22	0.1
*E. species*	406	1.9

## Data Availability

Data used to support the results of this study are included in the article.

## References

[1] Santajit S., Indrawattana N. (2016). Mechanisms of antimicrobial resistance in ESKAPE pathogens. Biomed. Res. Int..

[2] Laurentiu T.A., Nicoleta M., Octav P., Irina G., Marcela P., Otilia B., Corina C.V., Carmen M.C., Veronica L., Marutescu L. (2017). Resistance features of *Pseudomonas aeruginosa* strains isolated from patients with infectious complications of cardiovascular surgery. Biointerface Res. Appl. Chem..

[3] Fisher K., Phillips C. (2009). The ecology, epidemiology and virulence of *Enterococcus*. Microbiology.

[4] World Health Organization (2022). 2021 Antibacterial Agents in Clinical and Preclinical Development: An Overview and Analysis.

[5] World Health Organization (2020). Global Antimicrobial Resistance Surveillance System (GLASS) Report Early Implementation 2017–2018.

[6] Ahmed M.O., Baptiste K.E. (2018). Vancomycin-Resistant *Enterococci:* A Review of Antimicrobial Resistance Mechanisms and Perspectives of Human and Animal Health. Microb. Drug Resist..

[7] Rice L.B. (2008). Federal funding for the study of antimicrobial resistance in nosocomial pathogens: No ESKAPE. J. Infect. Dis..

[8] Suetens C., Latour K., Kärki T., Ricchizzi E., Kinross P., Moro M.L., Jans B., Hopkins S., Hansen S., Lyytikäinen O. (2018). Prevalence of healthcare-associated infections, estimated incidence and composite antimicrobial resistance index in acute care hospitals and long-term care facilities. Results from two European point prevalence surveys, 2016 to 2017. Eur. Surveill..

[9] Llaca-Díaz J.M., Mendoza-Olazarán S., Camacho-Ortiz A., Flores S., Garza-González E. (2012). One-year surveillance of ESKAPE pathogens in an Intensive Care Unit of Monterrey, Mexico. Chemotherapy.

[10] Lebreton F., Willems R.J.L., Gilmore M.S. (2014). *Enterococcus* Diversity, Origins in Nature, and Gut Colonization. Enterococci from Commensals to Leading Causes Drug Resistant Infection.

[11] Tacconelli E., Carrara E., Savoldi A., Harbarth S., Mendelson M., Monnet D.L., Pulcini C., Kahlmeter G., Kluytmans J., Carmeli Y. (2018). Discovery, research, and development of new antibiotics: The WHO priority list of antibiotic-resistant bacteria and tuberculosis. Lancet Infect. Dis..

[12] Shrivastava S.R., Shrivastava P.S., Ramasamy J. (2018). World health organization releases global priority list of antibiotic-resistant bacteria to guide research, discovery, and development of new antibiotics. JMS J. Med. Soc..

[13] CDC (2019). Antibiotic resistance threats in the United States. Atlanta (GA): Antibiotic Resistance Coordination and Strategy Unit within the Division of Healthcare Quality Promotion, National Center for Emerging and Zoonotic Infectious Diseases.

[14] O’Neill J. (2016). Tackling Drug-Resistant Infections Globally: Final Report and Recommendations.

[15] National Health Laboratory Services (2018). Standard Operating Procedures: Reporting of Notifiable medical conditions (NMC) version 2.0.

[16] Suppli M.R., Aabenhus Z.B., Harboe L.P., Andersen M., Tvede J.-U.S. (2011). Jensen. Mortality in enterococcal bloodstream infections increases with inappropriate antimicrobial therapy. Clin. Microbiol. Infect..

[17] Edmond M.B., Ober J.F., Dawson J.D., Weinbaum D.L., Wenzel R.P. (1996). Vancomycin-resistant enterococcal bacteremia: Natural history and attributable mortality. Clin. Infect. Dis..

[18] World Health Organization (2018). Surveillance Report of Antimicrobial Resistance and Consumption of Antibiotics in South Africa.

[19] García-Solache M., Rice L.B. (2019). The *Enterococcus*: A model of adaptability to its environment. Clin. Microbiol. Rev..

[20] O’Driscoll T., Crank C.W. (2015). Vancomycin-resistant enterococcal infections: Epidemiology, clinical manifestations, and optimal management. Infect. Drug Resist..

[21] Diekema D.J., Hsueh P.R., Mendes R.E., Pfaller M.A., Rolston K.V., Sader H.S., Jones R.N. (2019). The microbiology of bloodstream infection: 20-year trends from the SENTRY antimicrobial surveillance program. Antimicrob. Agents Chemother..

[22] De Kraker M.E.A., Jarlier V., Monen J.C.M., Heuer O.E., Van De Sande N., Grundmann H. (2013). The changing epidemiology of bacteraemias in Europe: Trends from the European Antimicrobial Resistance Surveillance System. Clin. Microbiol. Infect..

[23] Clinical and Laboratory Standards Institute (2019). Performance Standards for Antimicrobial Susceptibility Testing, 13th Information Supplement.

[24] Abera A., Tilahun M., Tekele S.G., Belete M.A. (2021). Prevalence, antimicrobial susceptibility patterns, and risk factors associated with enterococci among pediatric patients at Dessie Referral Hospital, Northeastern Ethiopia. BioMed. Res. Int..

[25] Alotaibi F.E., Bukhari E.E. (2017). Emergence of Vancomycin-resistant *Enterococci* at a teaching hospital, Saudi Arabia. Chin. Med. J..

[26] Gagetti P., Bonofiglio L., García Gabarrot G., Kaufman S., Mollerach M., Vigliarolo L., von Specht M., Toresani I., Lopardo H.A. (2019). Resistencia a los β-lactámicos en enterococos. Rev. Argent. Microbiol..

[27] Kristich C.J., Rice L.B., Arias C.A. (2014). Enterococcal infection-treatment and antibiotic resistance. Enterococci: From Commensals to Leading Causes of Drug Resistant Infection.

[28] European Centre for Disease Prevention and Control (2018). Surveillance of Antimicrobial Resistance in Europe-Annual Report of the European Antimicrobial Resistance Surveillance Network (EARS-Net) 2017.

[29] Olawale K.O., Fadiora S.O., Taiwo S.S. (2011). Prevalence of hospital acquired enterococci infections in two primary-care hospitals in Osogbo, Southwestern Nigeria. Afr. J. Infect. Dis..

[30] Ferede Z.T., Tullu K.D., Derese S.G., Yeshanew A.G. (2018). Prevalence and antimicrobial susceptibility pattern of *Enterococcus* species isolated from different clinical samples at Black Lion Specialized Teaching Hospital, Addis Ababa, Ethiopia. BMC Res. Notes.

[31] Abebe W., Endris M., Tiruneh M., Moges F. (2014). Prevalence of vancomycin resistant Enterococci and associated risk factors among clients with and without HIV in Northwest Ethiopia: A cross-sectional study. BMC Public Health.

[32] Solomon F.B., Wadilo F.W., Arota A.A., Abraham Y.L. (2017). Antibiotic resistant airborne bacteria and their multidrug resistance pattern at University teaching referral Hospital in South Ethiopia. Ann. Clin. Microbiol. Antimicrob..

[33] Toru M., Beyene G., Kassa T., Gizachew Z., Howe R., Yeshitila B. (2018). Prevalence and phenotypic characterization of *Enterococcus* species isolated from clinical samples of pediatric patients in Jimma University Specialized Hospital, south west Ethiopia. BMC Res. Notes.

[34] Billington E.O., Phang S.H., Gregson D.B., Pitout J.D., Ross T., Church D.L., Laupland K.B., Parkins M.D. (2014). 2 incidence, risk factors, and outcomes of enterococcus spp. blood stream 3 infections: A population-based study. Int. J. Infect. Dis..

[35] (2021). Surveillance Report for Antimicrobial Resistance and Consumption of Antimicrobials in South Africa. https://www.knowledgehub.org.za/elibrary/surveillance-antimicrobial-resistance-and-consumption-antimicrobials-south-africa-2021.

[36] Tedim A.P., Ruiz-Garbajosa P., Corander J., Rodríguez C.M., Cantón Willems R.J., Baquero F., Coque T.M. (2015). Population biology of intestinal *Enterococcus* isolates from hospitalized and nonhospitalized individuals in different age groups. Appl. Environ. Microbiol..

[37] Kapoor L., Randhawa V.S., Deb M. (2005). Antimicrobial resistance of enterococcal blood isolates at a pediatric care hospital in India. Jpn. J. Infect. Dis..

[38] Shrestha L.B., Baral R., Poudel P., Khanal B. (2019). Clinical, etiological and antimicrobial susceptibility profile of pediatric urinary tract infections in a tertiary care hospital of Nepal. BMC Paediatr..

[39] Anteneh A., Zeleke G., Demissie A., Setegn E. (2017). Antimicrobial resistance pattern of bacterial isolates from different clinical specimens in Southern Ethiopia: A three-year retrospective study. Afr. J. Bacteriol. Res..

[40] Miller W.R., Munita J.M., Arias C.A. (2014). Mechanisms of antibiotic resistance in enterococci. Expert Rev. Anti-Infect. Ther..

